# p53 exerts anticancer effects by regulating enhancer formation and activity

**DOI:** 10.7555/JBR.37.20230206

**Published:** 2024-05-29

**Authors:** Shuhan Chen, Xuchun Wang, Nan Yang, Yuechi Song, He Cheng, Yujie Sun

**Affiliations:** 1 Key Laboratory of Human Functional Genomics of Jiangsu Province, School of Basic Medical Sciences, Nanjing Medical University, Nanjing, Jiangsu 211166, China; 2 Department of Cell Biology, School of Basic Medical Sciences, Nanjing Medical University, Nanjing, Jiangsu 211166, China; 3 Jiangsu Key Lab of Cancer Biomarkers, Prevention and Treatment, Collaborative Innovation Center for Personalized Cancer Medicine, Nanjing Medical University, Nanjing, Jiangsu 211166, China

**Keywords:** p53, enhancer, tumor, malignant transformation

## Abstract

The abnormality of the p53 tumor suppressor is crucial in lung cancer development, because p53 regulates target gene promoters to combat cancer. Recent studies have shown extensive p53 binding to enhancer elements. However, whether p53 exerts a tumor suppressor role by shaping the enhancer landscape remains poorly understood. In the current study, we employed several functional genomics approaches to assess the enhancer activity at p53 binding sites throughout the genome based on our established *TP53* knockout (KO) human bronchial epithelial cells (BEAS-2B). A total of 943 active regular enhancers and 370 super-enhancers (SEs) disappeared upon the deletion of p53, indicating that p53 modulates the activity of hundreds of enhancer elements. We found that one p53-dependent SE, located on chromosome 9 and designated as *KLF4*-SE, regulated the expression of the Krüppel-like factor 4 (*KLF4*) gene. Furthermore, the deletion of p53 significantly decreased the *KLF4*-SE enhancer activity and the *KLF4* expression, but increased colony formation ability in the nitrosamines 4-(methylnitrosamino)-1-(3-pyridyl)-1-butanone-induced cell transformation model. Subsequently, in *TP53* KO cells, the overexpression of KLF4 partially reversed the increased clonogenic capacity caused by p53 deficiency. Consistently, *KLF4* expression also decreased in lung cancer tissues and cell lines. It appeared that overexpression of KLF4 significantly suppressed the proliferation and migration of lung cancer cells. Collectively, our results suggest that the regulation of enhancer formation and activity by p53 is an integral component of the p53 tumor suppressor function. Therefore, our findings offer some novel insights into the regulation mechanism of p53 in lung oncogenesis and introduce a new strategy for screening therapeutic targets.

## Introduction

The loss of p53 function is a hallmark of cancer development. The inactivation of wild-type (WT) p53 in normal cells weakens classical tumor suppressor effects, thereby facilitating cancer development. Lung cancer is the most commonly diagnosed cancer worldwide and the leading cause of cancer-related deaths. Approximately 60% of lung cancer cases harbor functional inactivation or mutations of p53^[1–2]^.

Previous studies have mainly focused on the functional role of p53 in regulating target gene promoters. However, recent analyses of the chromatin binding landscape have revealed that p53 not only binds to promoter regions but also non-coding regions with enhancer characteristics, implying its functional role in enhancer regulation^[3]^.

Enhancers are genomic elements that regulate the transcription of target genes over long distances by forming chromatin loops. Genome-wide analyses have revealed that enhancer deregulation, including the loss of anti-tumor enhancers and the formation of pro-tumor enhancers, occurs in multiple cancers^[4]^. Enhancers, as transcription factor binding sites, are characterized by specific chromatin modifications. Histone 3 lysine 4 monomethylation (H3K4me1) and histone 3 lysine 27 acetylation (H3K27ac) are commonly used as hallmarks to identify putative genome-wide enhancers^[5]^. Although H3K4me1 modification peaks indicate putative enhancer regions, active enhancers are defined by co-localized H3K4me1 and H3K27ac modification peaks. Recent studies have identified a new subset of enhancers called super-enhancers (SEs) that are clusters of adjacent enhancers in the genome. SEs regulate the expression of genes specifying cell identity and functionally conform to cell type-specific biological processes^[6]^. Compared with regular enhancers, SEs strongly activate gene expression at significantly higher levels^[6]^.

Recent studies demonstrated that the formation and activity of enhancers in the genome were influenced by the expression levels or activity of certain transcription factors^[7–8]^, and that transcription factors collaborated with chromatin-related modification factors to alter histone modifications or chromatin accessibility, thereby remodeling the enhancer landscape^[9]^. It is known that the reconfigured enhancer landscape alters gene expression, contributing to tumorigenesis. For example, Watt *et al*^[10]^ reported that an increased level of several activator protein-1 (AP-1) transcription factor proteins was implicated in the activation of many new enhancers to trigger cancer cell phenotypes. Sun *et al*^[11]^ also demonstrated that the overexpression of the transcription factor HOXA9 in myeloid and B progenitor cells induced the emergence of leukemia-specific enhancers and reprogramed the enhancer landscape by recruiting the transcription factor CCAAT enhancer binding protein alpha (CEBPa) and the myeloid/lymphoid or mixed lineage leukemia 3 (MLL3)/MLL4 complex, leading to the activation of an ectopic embryonic gene program and promoting leukemogenesis.

p53 is a well-known classical transcription factor whose binding sites are enriched in both promoter and promoter-distal enhancer regions. Previous investigations revealed that p53 binding to DNA induced considerable alterations in chromatin accessibility^[3,12]^. Therefore, proteins such as p53 may manipulate DNA and chromatin structures to establish a requisite order of transcription factor binding, inducing specific chromatin repressive or activated forms. These findings suggest that p53 may regulate enhancer activity, at least in part, by modulating chromatin accessibility.

In the current study, we aimed to investigate whether p53 suppressed tumors by regulating enhancer formation and activity. We established a *TP53* knockout (KO) human bronchial epithelial cell line (BEAS-2B) and used ChIP-seq and RNA-seq to screen for p53-dependent active enhancers and their target genes. We also investigated the role of Krüppel-like factor 4 (*KLF4*)-SE in lung cancer development and uncovered a novel function of p53 in regulating enhancers, suggesting a new strategy for identifying unknown targets.

## Materials and methods

### Cell culture

The BEAS-2B cell line was kindly provided by Professor Chaojun Li (Nanjing Medical University, Nanjing, China), the A549 cell line was kindly supplied by Professor Lin Xu (Jiangsu Cancer Hospital, Nanjing, China), and the H1703 cell line was kindly supplied by Professor Yonggang Zhao (Suzhou Institute of Systems Medicine, Suzhou, China). The culture medium used for BEAS-2B cells was DMEM, while the 1640 medium was used for H1703 and A549 cells. Both media were supplemented with fetal bovine serum (10%), penicillin (10^5^ U/L), and streptomycin (100 mg/L). Cells were passaged at a 1∶4 ratio and cultured in a CO_2_ incubator at a temperature of 37 ℃ with a CO_2_ concentration of 5%.

### CRISPR-Cas9 genome editing technology

The specific gene fragment was deleted in BEAS-2B cells by co-transfecting the single guide RNA (sgRNA) plasmid and Cas9 overexpression plasmid. The primers used in the construction of sgRNA plasmids are sgRNA-exon5F and sgRNA-exon5R, with their respective sequences detailed in ***Supplementary Table 1*** (available online). After confirming the deletion efficiency, single cells were sorted into separate 96-well plates using the Aria Ⅱ cell sorting machine (BD Biosciences, Franklin Lake, NJ, USA). Western blotting and sequencing (Anhui General Sequencing Company, Chuzhou, China) were performed to confirm the KO of the *TP53* gene.

### Western blotting

Protein extracts were separated on 10% polyacrylamide gels and transferred to PVDF membranes (Roche Applied Science, Basel, Switzerland). The membranes were then blocked for 1 h and incubated overnight with the following antibodies: anti-p53 (1∶5000; Cat. #sc-126x, Santa Cruz Biotechnology, Dallas, Texas, USA), anti-KLF4 (1∶2000; Cat. #CY6648, Abways Technology, Shanghai, China), anti- heat shock protein 90 (HSP90; 1∶2000; Cat. #A5006, Abclonal Technology, Wuhan, China), and anti-GAPDH (1∶8000; Cat. #MB001H, Bioworld Technology, Bloomington, MN, USA). Subsequently, the membranes were incubated with appropriate secondary antibodies at room temperature for 1 h. The signals of the bands were detected using ECL reagents (Cat. #180-501, Tanon, Shanghai, China).

### RNA isolation and real-time reverse transcription PCR (RT-qPCR)

The total RNA was isolated from cells using RNAiso Plus Reagent (Cat. #9019, Takara, Shiga, Japan). The cDNA was synthesized from 500 ng of total RNA using a reverse transcriptase cDNA synthesis kit (Cat. #R323-01, Vazyme, Nanjing, China). Subsequently, qPCR was performed using the SYBR Green PCR Kit (Cat. #Q321-02, Vazyme) and the 7500 Fast real-time PCR system (Applied Biosystems, Wilmington, DE, USA). The mRNA levels of *P21*, *KLF4*, and *IL7R* were normalized to *ACTB*. The primers used for qPCR are RT-*p21*-F, RT-*p21*-R, RT-*KLF4*-F, RT-*KLF4*-R, RT-*IL7R*-F, RT-*IL7R*-R, RT-*ACTB*-F, and RT-*ACTB*-R, with their respective sequences detailed in ***Supplementary Table 1***.

### Host-cell reactivation assay

The pGL3 promoter luciferase vector was treated with 12% H_2_O_2_ (v/v) for 1 h, and the damaged plasmid was purified through ethanol precipitation. The plasmid was then transfected into WT and *TP53* KO cells, and the DNA repair capacity was measured using a luciferase assay.

### Chromatin immunoprecipitation (ChIP) and cleavage under targets and release using nuclease (CUT&RUN) assays

For the ChIP assay, a ChIP assay kit (Cat. #17-610, Millipore, Burlington, MA, USA) was used. A total of 1 × 10^7^ cells for each sample were fixed with 1% formaldehyde at 37 ℃ for 10 min. The cells were lysed, sonicated into 100 to 500 bp fragments, and incubated with the antibody at 4 ℃ overnight. Cross-linking reversal was carried out at 65 ℃ for 5 h, followed by DNA isolation for subsequent sequencing. For the CUT&RUN assay, a Hyperactive pA-MNase for the CUT&RUN kit (Cat. #HD101-01, Vazyme) was used. A total of 5 × 10^5^ cells for each sample were fixed with ConA Beads Pro at 37 ℃ for 10 min. After overnight incubation with the primary antibody, DNA was fragmented and purified, and the p53 enrichment levels of *KLF4*-Enh1 (one of the activity enhancer elements located in *KLF4* super-enhancer region) and negative control were normalized to Spike in DNA and detected by qPCR. The primers used are qChIP-*KLF4*-Enh-F, qChIP-*KLF4*-Enh-R, qChIP-negative control-F, qChIP-negative control-R, Spike in DNA-F, and Spike in DNA-R, with their respective sequences detailed in ***Supplementary Table 1***. Antibodies used in these assays were the H3K4me1 antibody (Cat. #5326S, Cell Signaling Technology, Boston, MA, USA), H3K27ac antibody (Cat. #07-360, Millipore), and p53 antibody (Cat. #ab1101, Abcam, Cambridge, UK).

### ChIP-seq and RNA-seq data analysis

The ChIP-seq and RNA-seq libraries were sequenced on the Illumina sequencing platform by Novogene Co., Ltd. (Beijing, China). The sequencing data were compared with the reference genome hg38 of the Human genome. We used the Ubuntu system (version 22.04, Canonical), RStudio (version 4.2.2, RStudio), and TBtools software (version 2.019, China) to analyze the ChIP-seq and RNA-seq data. Additionally, we employed the Integrative Genomics Viewer (IGV) software to visualize the enrichment peaks of ChIP-seq. TBtools and Bedtools (version 2.28.0, Quinlan laboratory at the University of Utah) were used to analyze the differential peaks and generate Venn diagrams to screen for the active enhancers. Moreover, we used ChIPseeker (version 1.32.1, Bioconductor) to visualize the distribution of enrichment peaks in each chromatin. The enrichment peaks were classified and annotated according to the gene regions of the genome, including promoter, 5′ UTR, 3′ UTR, exon, intron, and intergenic, respectively. Furthermore, the rank ordering of SEs (ROSE) (version 1.3.1, Young Lab at Whitehead Institute for Biomedical Research) algorithm was used to identify SEs. Gene Ontology (GO, http://www.geneontology.org/) and gene set enrichment analysis (GSEA, https://www.gsea-msigdb.org/gsea) were utilized to classify and enrich gene functions. The Hi-C data of the IMR90 cell line (from GSE63525) were analyzed using the 3D genome browser (http://3dgenome.fsm.northwestern.edu/), and GEPIA (http://gepia.cancer-pku.cn/) data were visualized to compare the expression of the *KLF4* gene in lung cancer and normal tissues.

### Plasmid construction and luciferase reporter assay

The overexpression vector for human *KLF4* (pLVX-*KLF4*) was cloned from the full-length cDNA. The super-enhancer (SE) *KLF4*-SE and *IL7R*-Enh regions were cloned into the upstream of the SV40 promoter in the pGL3-promoter reporter vector respectively, which expressed the firefly luciferase for the luciferase assay. The primers used in the construction of plasmids are pLVX-*KLF4*-F and pLVX-*KLF4*-R, *KLF4*-Enh1-F, *KLF4*-Enh1-R, *IL7R*-Enh-F, and *IL7R*-Enh-R, with their respective sequences detailed in ***Supplementary Table 1***. For the luciferase reporter assay, cells were co-transfected with a series of plasmids, including the firefly reporter constructs containing the *KLF4*-SE, the Renilla-expressing plasmid, or the pGL3 promoter vector control plasmid. The firefly luciferase activity was measured using the Dual Luciferase Assay System (Promega, USA) 24 h after the transfection and normalized to the Renilla luciferase activity.

### Nitrosamines 4-(methylnitrosamino)-1-(3-pyridyl)-1-butanone (NNK) malignant transformation cell model

To induce malignant transformation of cells, the WT and *TP53* KO BEAS-2B cells were treated with 0.4 g/L (1.8 mmol/L) NNK (Cat. #78013, Sigma, Burlington, MA, USA) for 10 generations, while the control cells were treated with an equal volume of DMSO for 10 generations.

### Soft agar colony formation assay

Anchorage-independent cell growth was analyzed by plating 2 × 10^3^ cells mixed with the complete medium on a surface of 0.75% bottom agarose containing 0.25% top agarose. After 22 to 28 days of incubation, colonies were stained with the methylthiazolyldiphenyl-tetrazolium bromide solution and visually counted.

### Enhancer RNA (eRNA) knockdown by locked nucleic acid (LNA)-antisense oligonucleotides (ASO)

ASOs containing LNA modifications were synthesized by GenePharma (Shanghai, China). ASO-eRNA and ASO-negative control (NC) sequences used are provided in ***Supplementary Table 1***. As a negative control, an LNA-ASO sequence not targeting the human genome was employed for comparison. Following the transfection of 100 μmol/L ASO sequences into WT-DMSO_10th_ cells (WT cells treated by DMSO for 10 generations) for 48 h, RNA was extracted from the cells for subsequent RT-qPCR analysis. Quantification of *KLF4*-eRNA (RNA transcribed by *KLF4* enhancer) and *KLF4* mRNA levels was performed, with the results normalized to that of *ACTB*. The primers utilized for RT-PCR are *KLF4*-eRNA-F, *KLF4*-eRNA-R, RT-*KLF4*-F, RT-*KLF4*-R, RT-*ACTB*-F, and RT-*ACTB*-R, with their respective sequences detailed in ***Supplementary Table 1***.

### Cell proliferation assay

A CCK-8 Cell Counting kit (Cat. #311-01, Vazyme) was used to perform the cell proliferation assay. After 24 h of cell inoculation on a 96-well plate, the CCK-8 reagent was added to the culture and the cells were then incubated in the dark for 1 h. Subsequently, the absorbance value of each well was measured at a wavelength of 450 nm using an enzyme-labeled instrument (Molecular Devices, Silicon Valley, CA, USA).

### Cell scratch assay

Twenty-four hours after cell inoculation on a 6-well plate, sterilized 10 μL pipette tips were used to create scratches on the cell monolayer. The cells were then washed with PBS, the culture medium was refreshed, and photos were taken. After a 24-h incubation, the cells were photographed again. The images were captured and processed using the ImageJ software (http://cnij.imjoy.io/) to measure the intermediate blank area at 0 and 24 h. To determine the cell migration rate, we divided the difference in the measurements between the initial blank area (at 0 h) and the final blank area (at 24 h) by the initial blank area.

### Statistical analysis

The experiments were independently repeated at least three times to ensure the result's reproducibility. GraphPad 9.0 software was used for data processing and analysis. The quantitative data were expressed as mean ± standard error of the mean. Statistical analysis was performed using an unpaired Student's *t*-test. A *P*-value less than 0.05 was considered statistically significant.

## Results

### *TP53* KO in normal bronchial epithelial line

**Figure 1:**
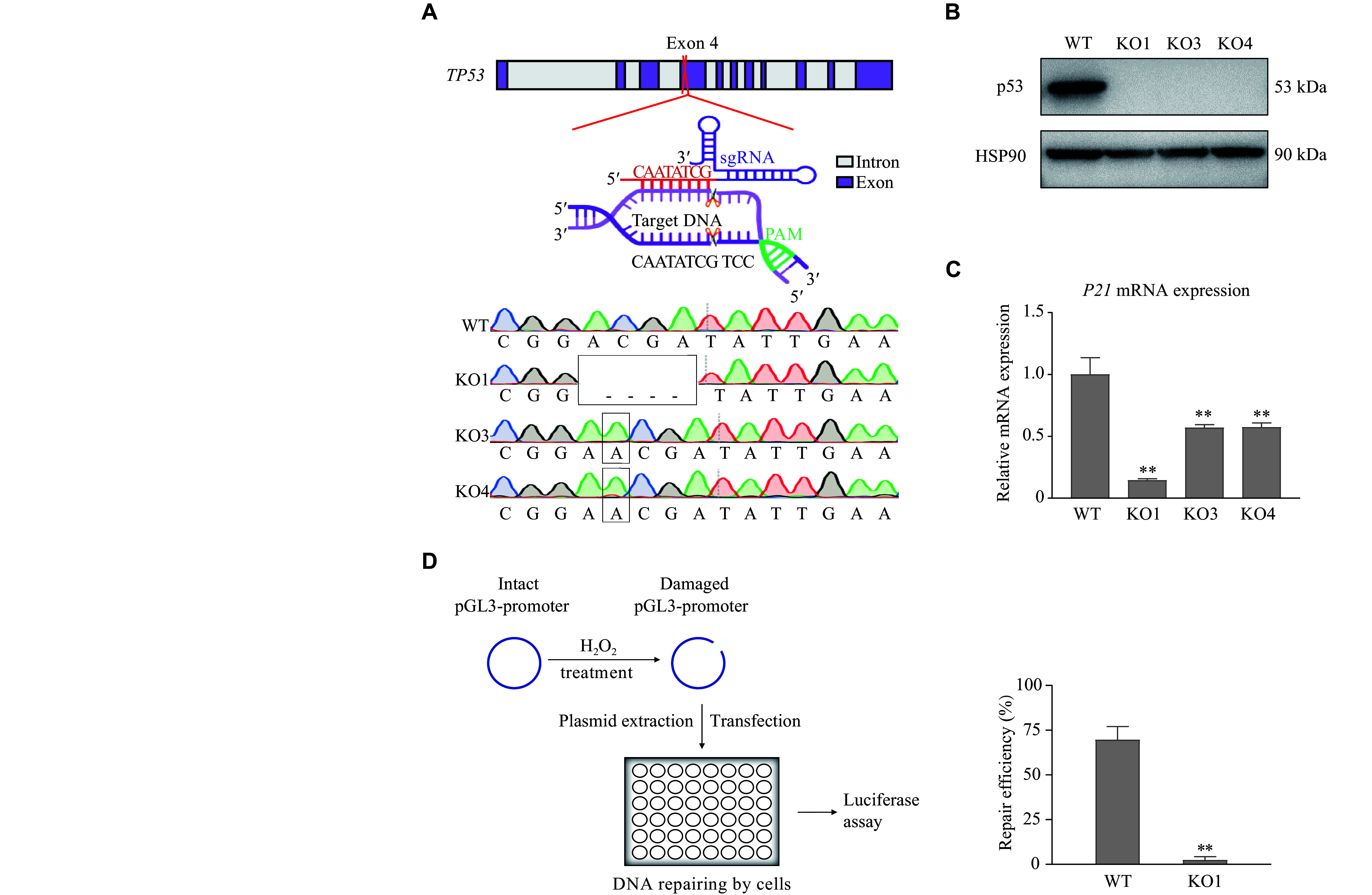
Establishment of *TP53* KO BEAS-2B cells.

To investigate the effect of p53 deletion on the enhancer landscape, we used CRISPR-Cas9 editing methods to generate diverse *TP53* KO BEAS-2B cells. As shown in the top panel of ***Fig. 1A***, the exon 4, which codes for the transcriptional activation domain of the p53 protein, was targeted. Sequencing (bottom panel, ***Fig. 1A***) and Western blotting (***Fig. 1B***) were performed to confirm the loss-of-function genotype in the derived clones. To verify the functional loss caused by *TP53* KO, RT-qPCR was performed to detect the expression of the canonical p53-target gene *P21* (***Fig. 1C***). The results showed that the expression of *P21* was reduced in all three *TP53* KO clones, compared with that of the WT BEAS-2B cells. Furthermore, a host-cell reactivation assay was performed in the *TP53* KO clones, and the results showed a significantly reduced DNA damage repair capacity in the *TP53* KO cells (***Fig. 1D***). These results demonstrated the successful establishment of *TP53* KO BEAS-2B cells.

### The loss of p53 triggered enhancer landscape remodeling

**Figure 2:**
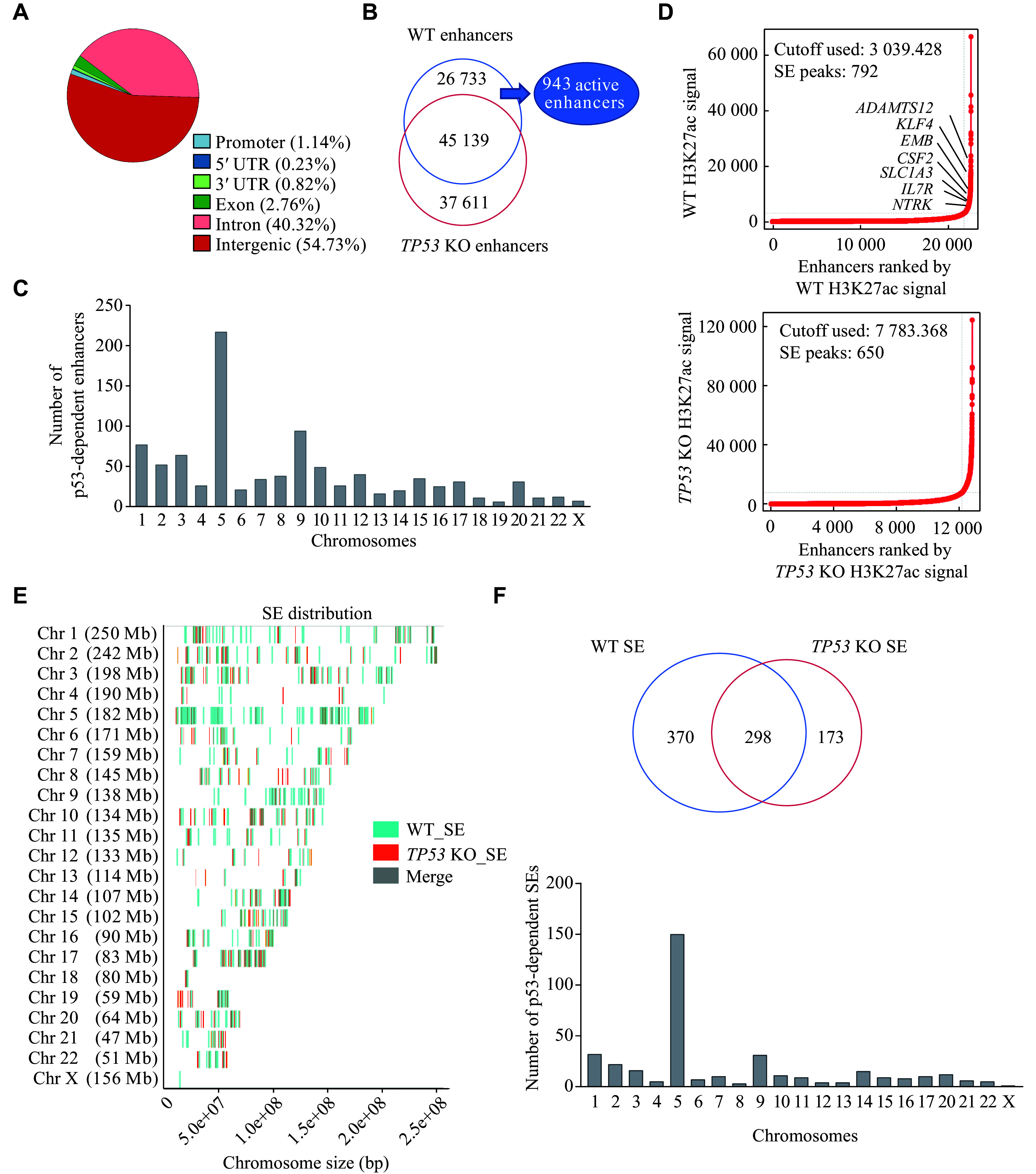
Loss of p53 leads to remodeling of the enhancer landscape.

To investigate the functional roles of p53 in regulating enhancer formation and activity, we performed the ChIP-seq assay. Firstly, we examined the genome-wide distribution of the p53 protein in WT BEAS-2B cells. The majority of p53 binding sites, approximately 95.05% of 11327 peaks, were located in intergenic or intronic regions within the BEAS-2B genome. In contrast, only about 4.95% of p53 peaks were found in gene promoters or other transcribed regions (*i.e.*, 5′ UTR, 3′ UTR, and exons) (***Fig. 2A***). We subsequently analyzed the alteration of the enhancer landscape following the p53 deletion by comparing the enrichment of H3K4me1 and H3K27ac in WT BEAS-2B cells with those in *TP53* KO BEAS-2B cells, which allowed us to identify the distribution of enhancers (H3K4me1) and active enhancers (H3K4me1 & H3K27ac). We identified 71872 enhancers in WT BEAS-2B cells, including 10007 active enhancers, while in *TP53* KO BEAS-2B cells, there were 82750 enhancers and 19111 active enhancers. Comparison of the H3K4me1 signatures between WT and *TP53* KO cells revealed a total of 26733 loss enhancers and 37611 gained enhancers following the *TP53* KO (***Fig. 2B***). Representative loci displaying the gained or lost H3K4me1 and H3K27ac are shown in ***Supplementary Fig. 1*** (available online). Among these 26733 loss enhancers, 943 were active enhancers that we referred to as p53-dependent enhancers (***Fig. 2B***). Distribution analysis of these 943 enhancers revealed an enrichment on chromosomes 5 and 9 (***Fig. 2C***). SEs were identified using the ROSE algorithm based on H3K27ac ChIP-seq signals (***Fig. 2D***). Interestingly, a similar distribution pattern was also observed in the SEs of WT BEAS-2B cells (***Fig. 2E***). Statistical analysis demonstrated that approximately half of the 370 p53-dependent SEs, which were lost with p53 deletion, were distributed on chromosomes 5 and 9 (***Fig. 2F***). These results indicated that the p53 deletion altered the landscape of enhancers as well as SEs in BEAS-2B cells. Notably, chromosomes 5 and 9 occupied the majority of p53-dependent enhancers and SEs, which were further investigated.

### Screening of downstream target genes of p53-dependent SEs on chromosomes 5 and 9

**Figure 3:**
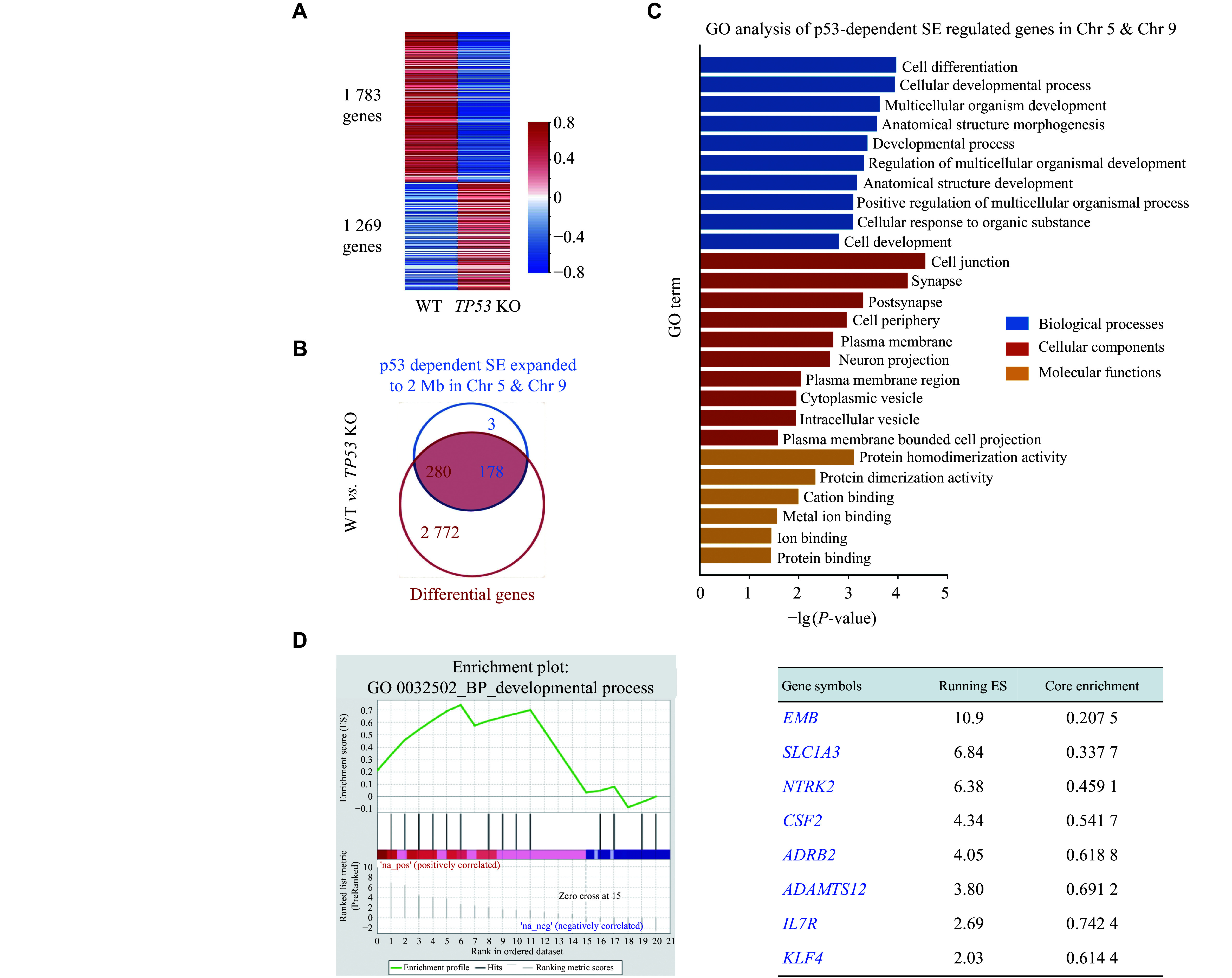
Screening of downstream target genes regulated by p53-dependent super-enhancers (SEs) on chromosomes 5 and 9.

To investigate the functional roles of p53-dependent SEs on chromosomes 5 and 9, we performed RNA-seq to screen for differentially expressed genes following the *TP53* KO. A total of 1269 genes were up-regulated, while 1783 genes were down-regulated because of the absence of p53 (***Fig. 3A***). Among these down-regulated genes, 280 genes were found within a 2-Mb range of the 178 p53-dependent SEs on chromosomes 5 and 9 (***Fig. 3B***), indicating their potential as target genes regulated by these enhancers. Furthermore, GO functional analysis revealed that p53-dependent SEs were predicted to regulate genes mainly governing cell differentiation and developmental process (***Fig. 3C***). GSEA was then performed for the differentially expressed genes associated with the developmental process located on chromosomes 5 and 9 (***Fig. 3D***). Notably, one of the top-scoring genes located on chromosome 9 is *KLF4*, a key transcription factor that regulates various cellular processes, such as cell growth, proliferation, and differentiation.

### Experimental confirmation of *KLF4*-SE as a p53-dependent SE

**Figure 4:**
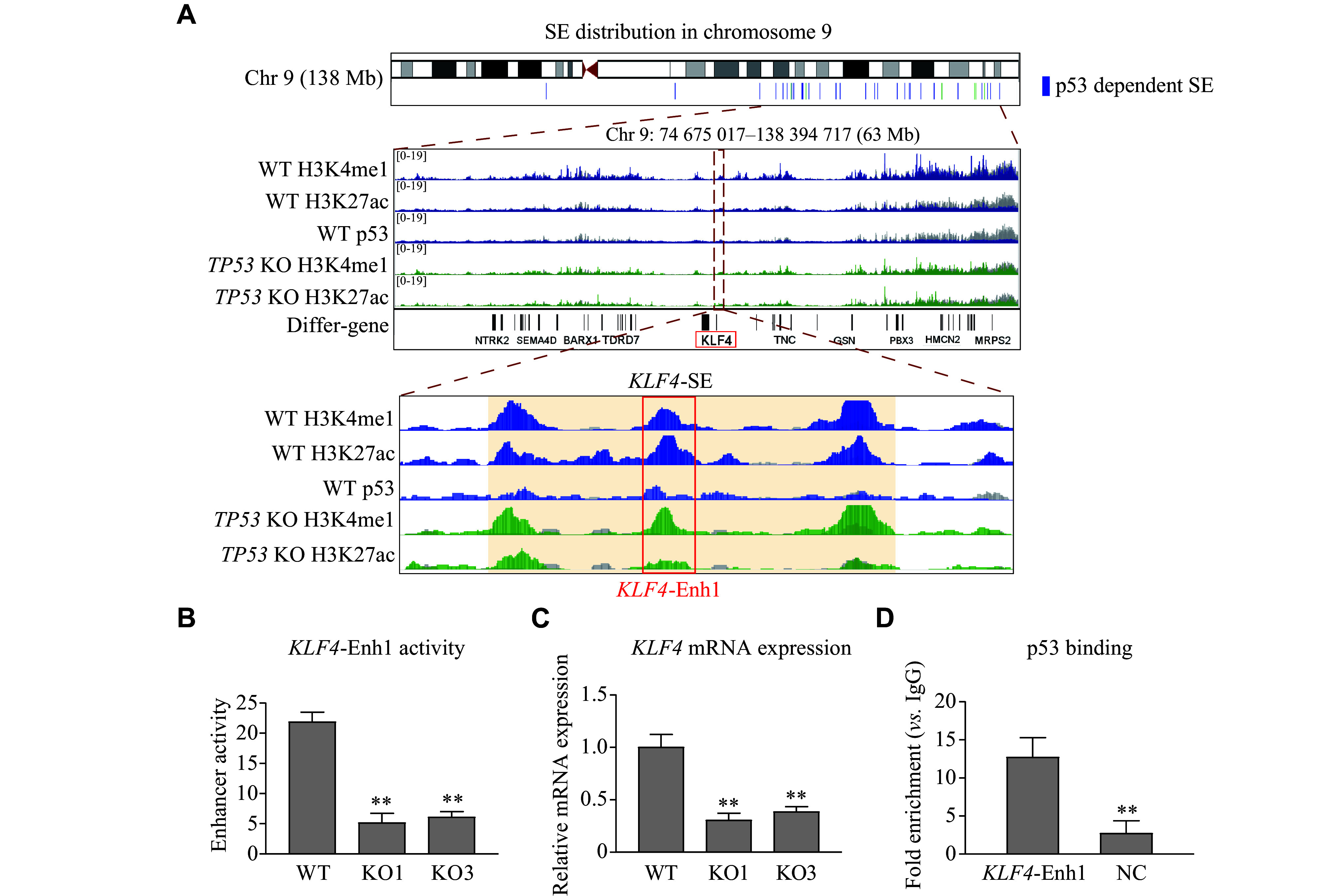
*KLF4*-SE is a p53-dependent super enhancer.

According to the ChIP-seq analysis, a p53-dependent SE, designated as *KLF4*-SE, was located at 262 kb upstream of the *KLF4* gene (***Fig. 4A***). The *KLF4*-SE spans a length of 5.9 kb and consists of three enhancer elements enriched in H3K4me1 and H3K27ac signatures. One of these enhancer elements (*KLF4*-Enh1) coexisted with p53 enrichment (***Fig. 4A***). Hi-C datasets (GSE63525) of IMR90 cells revealed that the *KLF4*-SE and *KLF4* promoter regions resided within the same topologically associating domains, indicating potential interactions between *KLF4*-SE and the *KLF4* promoter (***Supplementary Fig. 2***, available online). To validate the enhancer activity of *KLF4*-SE, we conducted a dual-luciferase reporter assay in WT BEAS-2B cells and two *TP53* KO BEAS-2B clones. The results showed that the enhancer activity in WT cells was approximately four times higher than that in the *TP53* KO cells, consistent with the expression pattern of *KLF4* in these cells (***Fig. 4B*** and ***4C***). The binding of p53 to the *KLF4*-Enh1 was also confirmed through the CUT&RUN assays, followed by qPCR (***Fig. 4D***). These results indicated that *KLF4*-SE functioned as a p53-dependent SE.

### *KLF4* was regulated by *KLF4*-SE during cell malignant transformation induced by NNK

**Figure 5:**
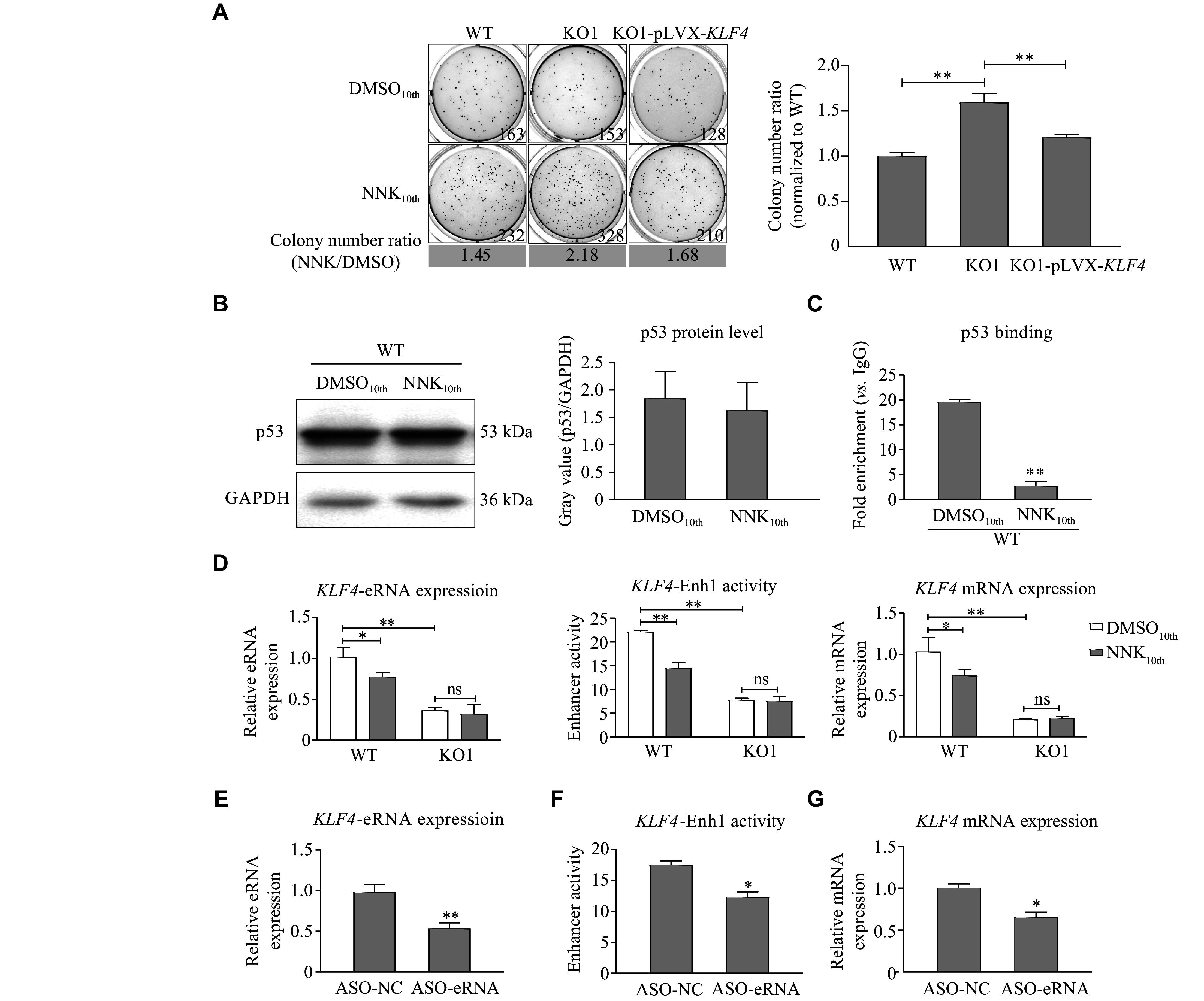
Regulation of *KLF4* by *KLF4*-SE during NNK-induced cell malignant transformation.

To evaluate biological significance of the p53-dependent enhancer *KLF4*-SE, we established the cell malignant transformation model by treating WT and *TP53* KO BEAS-2B cells with 0.4 g/L potent lung cancer-specific carcinogen NNK for 10 generations. To detect the cell malignant transformation ability, we conducted the soft-agar clone formation assay on WT and *TP53* KO BEAS-2B cells that had been treated with NNK or DMSO control. The results showed that the deletion of p53 caused an increase in colony formation after the NNK treatment. Moreover, the overexpression of KLF4 in *TP53* KO cells partially reversed the increased clonogenic capacity caused by the p53 deficiency (***Fig. 5A*** and ***Supplementary Fig. 3*** [available online]).

Compared with the DMSO control group, the binding of p53 to *KLF4*-Enh1 was significantly decreased after treatment with 0.4 g/L NNK for 10 generations, as determined by the CUT&RUN RT-qPCR assay. However, p53 expression remained unchanged as observed by Western blotting analysis (***Fig. 5B*** and ***5C***). Notably, the treatment with NNK resulted in a significant reduction in the levels of *KLF4*-Enh1 eRNA (*KLF4*-eRNA), the activity of the *KLF4*-Enh1 enhancer, and the expression of *KLF4* (***Fig. 5D***), which were consistent with the observed decline in p53 enrichment.

Given the roles of eRNA in mediating interactions between enhancers and promoters, we further investigated the effect of *KLF4*-Enh1 activity on the expression of downstream genes by using LNA-ASO sequences to interfere with *KLF4*-eRNA (***Fig. 5E***). Both the activity of *KLF4*-Enh1 and the expression levels of the downstream target gene *KLF4* were decreased (***Fig. 5F*** and ***5G***). In summary, these results highlighted the crucial role of *KLF4*-SE in regulating KLF4 expression during the NNK-induced cell malignant transformation.

### Overexpression of KLF4 reduced cell proliferation and migration in lung cancer cells

**Figure 6:**
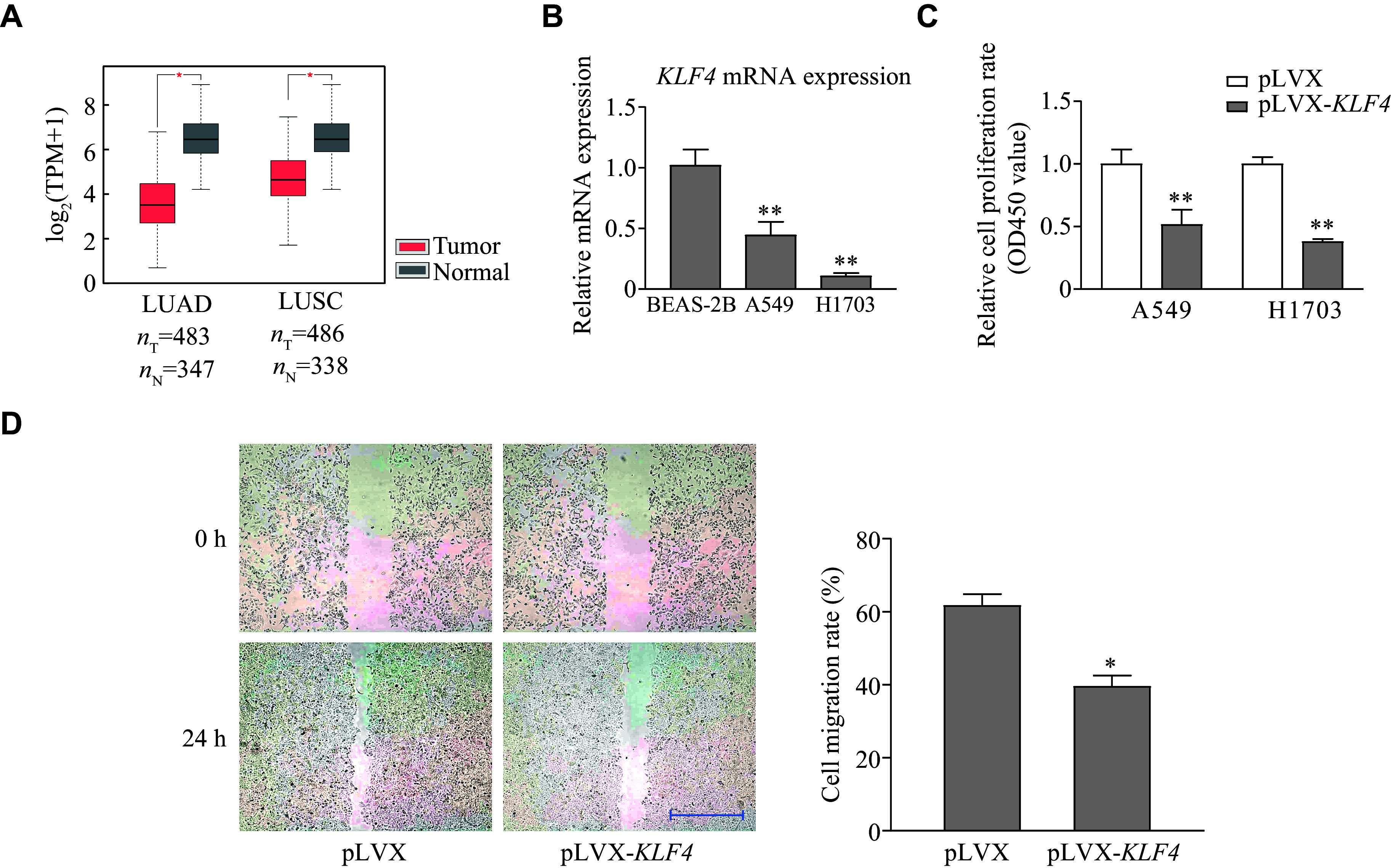
Overexpression of KLF4 reduces cell proliferation and migration in lung cancer cells.

To investigate the function of KLF4 during carcinogenesis, we first examined the expression of *KLF4* in lung cancer tissues. According to the GEPIA database, *KLF4* expression was significantly reduced in lung adenocarcinoma (LUAD) and lung squamous carcinoma (LUSC) (***Fig. 6A***). Next, we measured the expression of *KLF4* in human normal lung bronchial cell line BEAS-2B and lung cancer cell lines A549 and H1703. A significant reduction of *KLF4* mRNA expression was observed in these two lung cancer cell lines (***Fig. 6B***). Moreover, overexpression of KLF4 in the A549 and H1703 lung cancer cell lines resulted in a decline in cell proliferation as determined by the CCK-8 assay (***Fig. 6C***). Additionally, we observed a reduction in cell migration ability in A549 cells after KLF4 overexpression using the wound healing assay (***Fig. 6D***). These results indicated that KLF4 inhibited malignant phenotypes in lung cancer cells.

## Discussion

The tumor suppressor p53 is a well-characterized transcription factor that functions as a primary defense against genomic instability, and its functional loss plays a crucial role in the development and progression of lung cancer (***Supplementary Fig. 4***, available online).

Chromatin-binding profiles reveal specific interactions of p53 not only with promoter regions of nearby target genes but also with distant locations that harbor characteristics of enhancer domains^[13]^. In the current study, we designed and performed the ChIP-seq analysis to measure the accumulation of H3K4me1 and H3K27ac, evaluating the remodeling of the enhancer landscape after the deletion of p53. We detected 71872 enhancers in WT BEAS-2B cells, including 10007 active enhancers, while there were 82750 enhancers and 19111 active enhancers in *TP53* KO BEAS-2B cells. The comparison between the H3K4me1 and H3K27ac signatures in WT and *TP53* KO cells revealed a total of 943 p53-dependent enhancers and 370 p53-dependent SEs. These enhancers and SEs lose their enhancer features caused by the p53 deletion, which indicates that the presence of the key transcription factor p53 is sufficient to transform sequences into functional enhancer elements. Thus, we hypothesize that the background level of the p53 protein functions as a "monitor" of normal cells, partially by binding to and recruiting histone-modified proteins and promoting chromatin remodeling to form enhancers or regulate enhancer activation. The p53-modifying enzymes and tissue-specific co-transcription factors may possess inherent selectivity towards p53 molecules that are bound to specific sites in the genome and form enhancers. Therefore, the p53 protein appears to function as a key factor that modulates chromatin accessibility in healthy lung cells, leading to the enhancer landscape remodeling.

Several studies have also found that there are p53 binding sites within regulatory enhancer elements in different cells. For example, Korkmaz *et al*^[14]^ performed a comprehensive genetic screen in human fibroblast cells (BJ) to identify p53-bound enhancers required for oncogene-induced senescence by using a CRISPR-Cas9 sgRNA library, and identified a novel enhancer that might regulate the *CDKN1A* expression. Younger *et al*^[3]^ also found that the majority of sequences examined displayed p53-dependent enhancer activity during the DNA damage response in human fetal fibroblast GM06170 cells. Interestingly, most of the enhancers, especially SEs identified in the current study do not overlap with the p53 regulatory enhancers obtained from other studies. This suggests that the p53-dependent enhancers and SEs obtained in the current study exhibit lung tissue specificity. These findings may reflect that the tissue-specific co-transcription factors and/or the distinct preferences of co-transcription factors in different tissue environments may aid p53 in forming enhancers in different regions. Our findings uncover an important role for p53 in the modulation of enhancer landscape in lung cells.

KLF4 is an evolutionarily conserved zinc finger-containing transcription factor that regulates diverse cellular processes, such as cell growth, proliferation, and differentiation^[15–16]^. It was initially recognized as one of four factors involved in the induction of pluripotent stem cells, and subsequently, more and more studies have revealed the functional roles of KLF4 in tumorigenesis. In lung cancer, it was demonstrated that *KLF4* functioned as a tumor suppressor gene, the expression of KLF4 was down-regulated in the majority of primary lung cancers, and ectopic expression of KLF4 suppressed lung cancer cell proliferation, clonogenic formation, and tumor growth^[17–18]^. One study reported that over-expression of KLF4 in lung cancer cells inhibited cell migration and invasion^[19]^. These findings consistently support our results that KLF4 reduced cell proliferation and migration in lung cancer cells.

The p53-KLF4 pathway may involve a feedback loop, as evidenced in a study reporting that p53 acted as a regulatory factor for KLF4, while being regulated by KLF4 itself^[15]^. Akaogi *et al*^[20]^. demonstrated that inhibiting p53 expression in MCF-7 breast cancer cells led to the downregulation of *KLF4* mRNA levels, aligning with our findings. However, the specific underlying mechanism behind this regulation was not explored in their study. Additionally, previous research showed that KLF4 enhanced the DNA-binding affinity of p53 by facilitating the formation of a loosely arranged ternary complex on DNA^[21]^, and played a crucial role as an essential mediator of p53 in the transcriptional induction of *P21*^WAF1/Cip1[22]^. Whether KLF4 influences the regulation of p53 on the enhancer remains unclear with a lack of relevant research. However, based on previous findings, it is reasonable to hypothesize that *KLF4* may promote p53 regulation of its downstream enhancers, which warrants future investigation.

We found that the majority of p53-dependent enhancers and SEs were accumulated on chromosomes 5 and 9, which suggests an important role of chromosomes 5 and 9 in the p53-related regulation pathways. The chromosome 5p15.33 region has been identified as one of the independent factors associated with lung cancer in several large, collaborative, genome-wide association studies^[23]^. The deletion or downregulation of several tumor suppressor genes located on chromosome 9 has also been observed in lung cancer^[24–25]^. These suggest the important role of chromosomes 5 and 9 in lung cancer development.

Further GO analysis of the p53-dependent SEs located on chromosomes 5 and 9 showed that the functions of the target genes were primarily related to cell differentiation and developmental processes associated with carcinogenesis. The GSEA on the development process indicated that *IL7R*, *ADAMTS12*, and *ADRB2* might also significantly contribute to the energy score of this gene set, except for *KLF4*. Several studies have reported the associations between these genes and lung cancer. For example, one study reported that *IL7R* was a beneficial prognostic marker for patients with LUAD, and that its expression was positively correlated with both overall and progression-free survival rates in patients with LUAD and negatively correlated with tumor size, suggesting that IL-7R may inhibit the growth of tumor cells by affecting the percentage of infiltrating cells in the tumor immune microenvironment^[26]^. Another study showed that IL7R promoted the sensitivity of non-small cell lung cancer (NSCLC) cells by activating the IL-7R-JAK3/STAT5 signaling pathway to cisplatin, and inhibited tumor growth^[27]^. In the current study, we also examined the p53-dependent enhancer, IL7R-Enh, and its downstream target gene, *IL7R*. We observed that upon *TP53* KO, the enhancer activity of IL7R-Enh was significantly diminished, leading to a significant decrease in the expression of its target gene, *IL7R* (***Supplementary Fig. 5***, available online).

For *ADAMTS12*, one study suggested a protective effect of ADAMTS-12 against bronchial inflammation and hyper-responsiveness^[28]^. Another study reported that *Adamts12*^−/−^ mice exhibited a five-fold increase in the risk of developing lung cancer, demonstrating the role of *ADAMTS12* as a tumor suppressor gene in lung cancer^[29]^.

For *ADRB2*, one study reported that it was likely to be an important regulator of airway smooth muscle tone, because high levels of ADRB2 were associated with reduced lung function, asthma, and COPD^[30]^. Furthermore, ADRB2 expression was significantly decreased in LUAD tissues^[31]^, and patients with higher expression levels of ADRB2 had an increased overall survival rate in lung cancer^[31–32]^. Overall, the results of these studies on IL-7R, ADAMTS12, and ADRB2 indicate their anti-tumor functions in lung cancer.

Previous studies on p53 and lung cancer primarily focused on p53 mutational landscape, expression patterns, and functional implications in lung cancer^[33–34]^. In particular, these studies have predominantly investigated the binding and transcriptional activation of downstream genes in promoter regions to investigate the regulation of genes by p53. In the current study, we adopted a novel approach by investigating the role of p53-regulated enhancer elements involved in malignant transformation of lung cells, and we found a series of p53 downstream target genes throughout the entire genome, providing a basis for expanding the repertoire of p53 downstream genes and identifying new tumor suppressor genes for future studies of other cancers. Targeting these genes or their enhancers with specific activators may potentially achieve anticancer effects. Additionally, we also identified a few genes whose enhancers were negatively regulated by p53, often playing oncogenic roles. Exploiting these enhancers or eRNAs may be a promising approach for targeting downstream gene expression status and intervention, achieving tumor inhibition. This provides novel insights for drug development in the treatment of cancer.

In summary, p53 was found to play a pivotal role in shaping the enhancer landscape in normal lung cells, and the deletion of p53 led to a subset of enhancers and SEs losing their enhancer features. Interestingly, most of these p53-dependent SEs were enriched on chromosomes 5 and 9. We demonstrated the functions of *KLF4*-SE, one of the p53-dependent SEs, and further studied its anti-tumor role in the NNK model and lung cancer cells. These results suggest that p53 may exert the cancer suppressor role by changing the formation and activity of p53-dependent enhancers to regulate the downstream target genes. The current study provides a novel clue for understanding the p53 regulation mechanism in lung cancer carcinogenesis and a new strategy to screen for new therapeutic targets in lung cancer.

## SUPPLEMENTARY DATA

Supplementary data to this article can be found online.
