# Supplementary material for: p53 exerts anticancer effects by regulating enhancer formation and activity
Source: J Biomed Res. 2024 May 29;38(4):334–47. doi: 10.7555/JBR.37.20230206 (PMC11300520; doi:10.7555/JBR.37.20230206)
Supplement: Supplementary file 1 — Supplementary data to this article can be found online. [file jbr-38-4-334-S1.pdf]

Available online at www.jbr-pub.org.cn

Open Access at PubMed Central

Journal of Biomedical Research, 2024 0(000): 1–4

Supplementary Data

JBR

p53 exerts anticancer effects by regulating enhancer formation and activity

Shuhan Chen[1,2], Xuchun Wang[1,2], Nan Yang[1], Yuechi Song[2], He Cheng[1,2,✉], Yujie Sun[1,2,3,✉]

[1]*Key Laboratory of Human Functional Genomics of Jiangsu Province, School of Basic Medical Sciences, Nanjing Medical University, Nanjing, Jiangsu 211166, China;*
[2]*Department of Cell Biology, School of Basic Medical Sciences, Nanjing Medical University, Nanjing, Jiangsu 211166, China;*
[3]*Jiangsu Key Lab of Cancer Biomarkers, Prevention and Treatment, Collaborative Innovation Center for Personalized Cancer Medicine, Nanjing Medical University, Nanjing, Jiangsu 211166, China.*

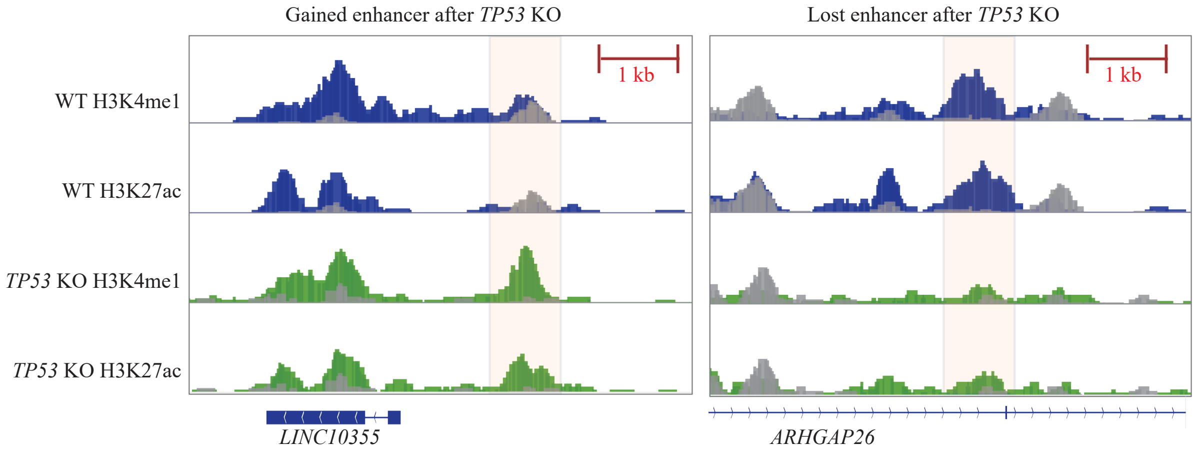

Supplementary Fig. 1 **Representative loci showing the gained or lost H3K4me1 and H3K27ac signature.** The blue and green peaks represent the genomic localization and trajectory of p53 binding events and histone modifications (H3K4me1 and H3K27ac) in wild-type (WT) and *TP53* knockout (KO) BEAS-2B cells, respectively. The yellow area represents the region of enhancers that have been obtained/disappeared.

✉Corresponding authors: He Cheng and Yujie Sun, Department of Cell Biology, School of Basic Medical Sciences, Nanjing Medical University, 101 Longmian Avenue, Nanjing, Jiangsu 211166, China. E-mails: chenghe@njmu.edu.cn (Cheng) and yujiesun@njmu.edu.cn (Sun).

Received: 01 September 2023; Revised: 08 February 2024; Accepted: 05 March 2024; Published online: 07 March 2024

CLC number: R73, Document code: A

The authors reported no conflict of interests.

This is an open access article under the Creative Commons Attribution (CC BY 4.0) license, which permits others to distribute, remix, adapt and build upon this work, for commercial use, provided the original work is properly cited.

© 2024 by Journal of Biomedical Research.

https://doi.org/10.7555/JBR.37.20230206

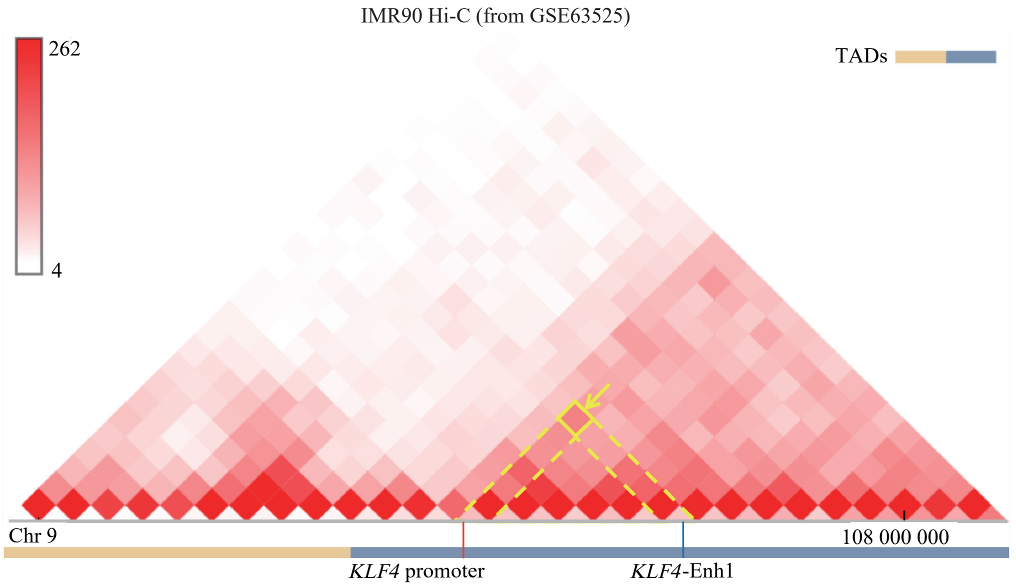

Supplementary Fig. 2 **Visualization of the reciprocal interaction strength between *KLF4*-Enh1 and the *KLF4* promoter in IMR90 cells measured by Hi-C.** Data were obtained from the GEO database (GSE63525).

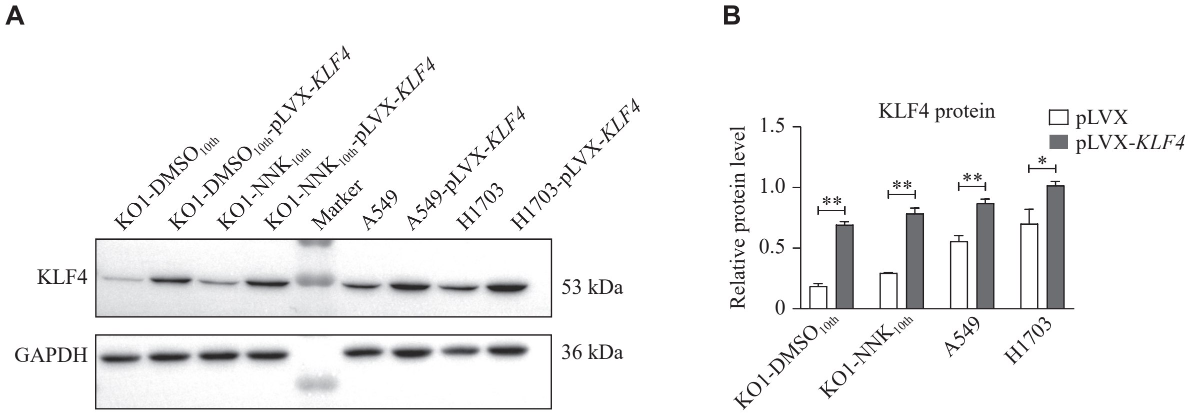

Supplementary Fig. 3 **Western blotting analysis of KLF4 overexpression in various cell lines.** A: Western blotting analysis of p53 levels in KO1-DMSO$_{10th}$, KO1-NNK$_{10th}$, A549, and H1703 cells. B: Grayscale analysis of Western blotting results (n = 3 per group). $^{*}P < 0.05$ and $^{**}P < 0.01$ by two-tailed unpaired Student's t-test.

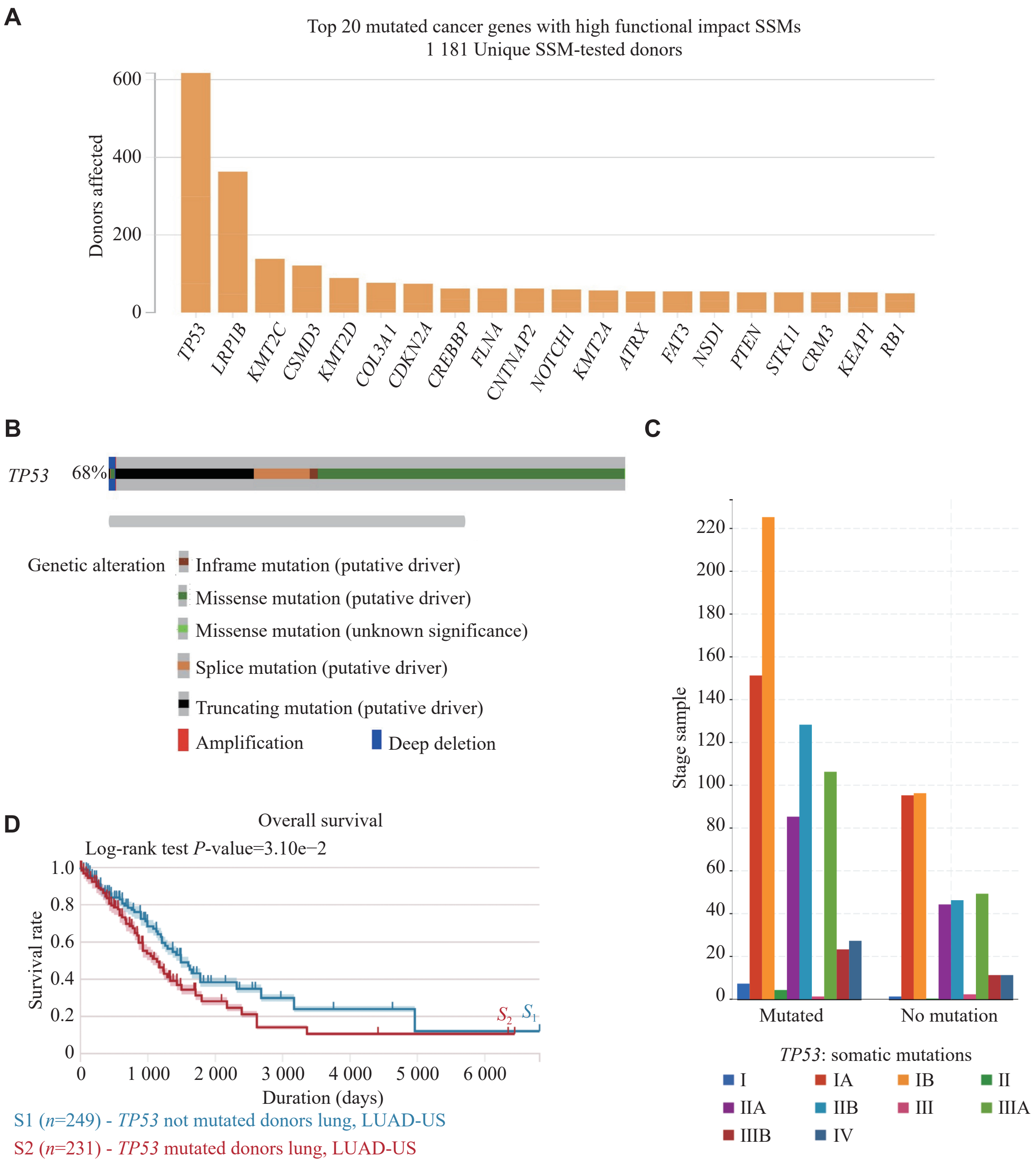

Supplementary Fig. 4 ***TP53*** **was the most frequently mutated cancer gene in cancer, and its mutation was associated with a poor prognosis in lung cancer.** A: Top 20 cancer genes with high functional impact somatic single mutations (SSMs) from the International Cancer Genome Consortium (ICGC) database. Among the 1 181 lung cancer cases analyzed, 617 cases have mutations in *TP53*. B: The Cancer Genome Atlas (TCGA) data from the cBioPortal database shows the types of *TP53* mutations in cancer patients. A total of 68% of lung cancer patients have *TP53* mutations (776/1144), including missense mutations, deletions, and copy number variations. C: Bar chart displaying the number of *TP53* mutation cases in patients with different stages of lung tumors. Data were obtained from the cBioPortal database. D: Survival analyses of lung cancer patients with or without *TP53* mutations. Data were downloaded from the ICGC database.

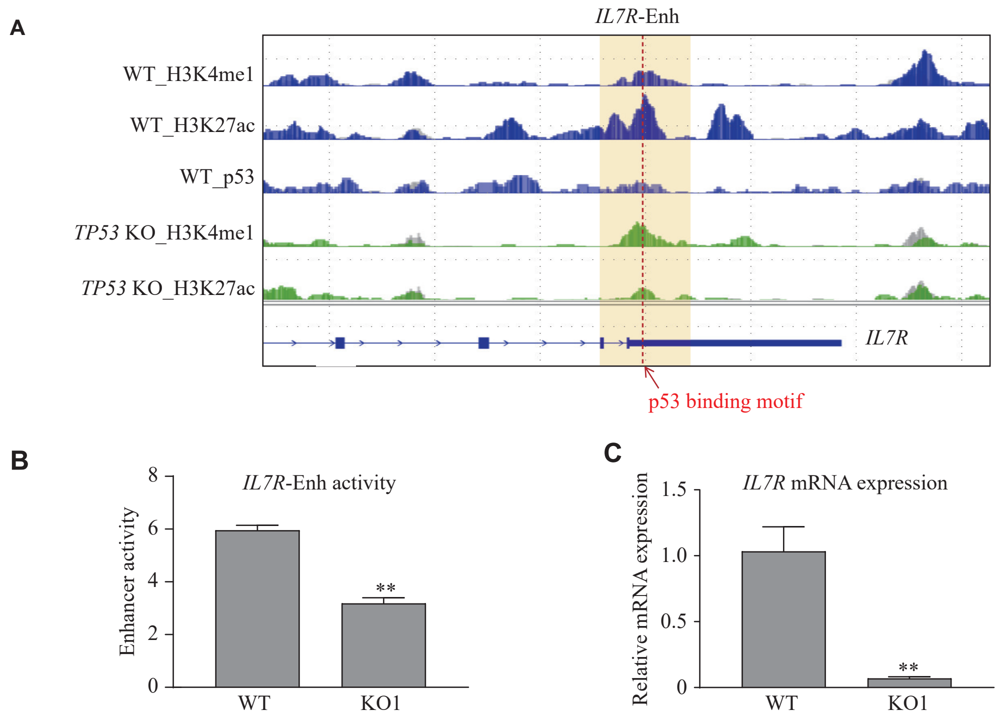

Supplementary Fig. 5 **p53-dependent enhancer IL7R-Enh and its target gene *IL7R* were positively regulated by p53.** A: Genomic location and tracks in the IL7R-Enh region, including p53 binding events and histone modifications (H3K4me1 and H3K27ac) of wild type (WT) (blue) and *TP53* knockout (KO) (green) BEAS-2B cells. The yellow region indicates enhancer IL7R-Enh, and the red dashed line represents the p53 binding motif. B: IL7R-Enh enhancer activity was measured by using a dual-luciferase assay in WT BEAS-2B cells and *TP53* KO1 clone. C: *IL7R* mRNA expression levels were detected by real-time reverse transcription PCR in WT BEAS-2B cells and *TP53* KO1 clone. The quantitative data were expressed as the mean ± standard error of the mean (n = 3 per group). $^{**}P < 0.01$ by the two-tailed unpaired Student's t-test.

Supplementary Table 1 **Primer sequences**

Primer name	Sequence（5′→3′）
sgRNA-exon5F	CACCGCGGCACCCGCGTCCGCGCCA
sgRNA-exon5R	AAACTGGCGCGGACGCGGGTGCCGC
RT-*p21*-F	ATGGAACTTCGACTTTGTCACC
RT-*p21*-R	AGGTCCACATGGTCTTCCTCT
RT-*KLF4*-F	CCCACATGAAGCGACTTCCC
RT-*KLF4*-R	CAGGTCCAGGAGATCGTTGAA
RT-*IL7R*-F	CCCTCGTGGAGGTAAAGTGC
RT-*IL7R*-R	CCTTCCCGATAGACGACACTC
RT-*ACTB*-F	TCATGAAGTGTGACGTGGACAT
RT-*ACTB*-R	CTCAGGAGGAGCAATGATCTTG
qChIP-*KLF4*-Enh-1F	AGAGCAGCCAGCAGACATTT
qChIP-*KLF4*-Enh-1R	CTTGTGAGCAGAGACAGGGG
qChIP-negative control-F	CCATCTCCCCCTCCCTCTC
qChIP-negative control-R	CTTGAGGCTCCCAGTTCACG
Spike in DNA-F	GCCTTCTTCCCATTTCTGATCC
Spike in DNA-R	CACGAATCAGCGGTAAAGGT
pLVX-*KLF4*-F	CGGAATTCATGAGGCAGCCACCTGGCGA
pLVX-*KLF4*-R	GCTCTAGATTAAAAATGCCTCTTCATGT
KLF4-Enh1-F	GGGGTACCTCAACAATGTTGCCAATGAA
KLF4-Enh1-R	TCCCCCGGGATTTGGACAGGTCCCATCT
IL7R-Enh-F	GGGGTACCCGATCATAAGAAGACTCTGGAACA
IL7R-Enh-R	CCGCTCGAGCCCTTTTAAATCATCTTTGTCGCT
KLF4-eRNA-F	TAGGATATGCCAGAAGTTTTCTCTT
KLF4-eRNA-R	GTCTCCGTTTCAATGACAAA
ASO-eRNA	GTCTCCGTTTCAATGACAAA
ASO-NC	GCGUATTATAGCCGATTAAC
